# Gene Feature Extraction Based on Nonnegative Dual Graph Regularized Latent Low-Rank Representation

**DOI:** 10.1155/2017/1096028

**Published:** 2017-03-30

**Authors:** Guoliang Yang, Zhengwei Hu

**Affiliations:** School of Electrical Engineering and Automation, Jiangxi University of Science and Technology, Ganzhou 341000, China

## Abstract

Aiming at the problem of gene expression profile's high redundancy and heavy noise, a new feature extraction model based on nonnegative dual graph regularized latent low-rank representation (NNDGLLRR) is presented on the basis of latent low-rank representation (Lat-LRR). By introducing dual graph manifold regularized constraint, the NNDGLLRR can keep the internal spatial structure of the original data effectively and improve the final clustering accuracy while segmenting the subspace. The introduction of nonnegative constraints makes the computation with some sparsity, which enhances the robustness of the algorithm. Different from Lat-LRR, a new solution model is adopted to simplify the computational complexity. The experimental results show that the proposed algorithm has good feature extraction performance for the heavy redundancy and noise gene expression profile, which, compared with LRR and Lat-LRR, can achieve better clustering accuracy.

## 1. Introduction

With the accelerated pace of modern life, the high incidence of cancer has brought great challenges to human health. How to detect, prevent, and treat cancer effectively has become an international hotspot of medical research. Gene expression profile is a specific cDNA sequence data of cells, which can describe cells' current physiological function and state. Researches show that tumor cells and normal cells could be identified effectively by analyzing and processing the original gene expression data. However, the scale of the gene expression profile is huge and complex due to the diversity and specificity of the cells; therefore the traditional methods of data analysis and processing have been unable to adapt to these extremely large-scale data.

Gene expression profile extracting includes two kinds of methods: linear and nonlinear. Early linear transformation methods include principal component analysis [1–3] (PCA), linear discriminant analysis [4–6] (LDA), and independent component analysis [7, 8] (ICA). The main methods of nonlinear transformation include kernel method [9], neural network [10, 11], manifold learning [12, 13], and sparse representation [14, 15]. In recent years, LRR [16–18] and neural networks have been widely used in feature extraction and classification of gene expression profile. Reference [19] used NMF for gene feature extraction and achieved more satisfactory results. Ref. [20] proposed a gene expression profile classification means based on ontology perception. Ref. [21] proposed a subcellular cooccurrence matrix feature extraction method. Ref. [22] proposed a gene expression profile classification method by neural network hybrid back-propagation. Ref. [23] proposed a supervised way of tumor prediction with multiview.

The size of the gene expression profile is large, and there are interrelationships between the samples. The internal spatial structure of the data may be destroyed in the process of linear transformation. In this paper, a model of feature extraction based on NNDGLLRR is proposed on the basis of Lat-LRR, which with low-rank sparse constraint can remove the redundant components of gene expression and suppress the noise. Nonnegative constraints make the calculation with a certain degree of sparsity, in line with the practical significance of the data, and enhance the robustness of the algorithm. And the manifold regularized constraint is introduced, so that the result of feature extraction can describe the spatial structure of the original data more completely.

## 2. Related Work

### 2.1. LRR

LRR is a combination of matrix low-rank decomposition and sparse decomposition. In recent years, it has been widely used in subspace clustering. LRR assumes that the original data comes from different subspaces and performs feature extraction by trying to find the lowest rank representation of the original data. And this low-rank representation coefficient is the reflection of the original data in the spatial distribution of structural information. If the original data **X** = [*x*_1_, *x*_2_, *x*_3_,…, *x*_*n*_] ∈ *ℝ*^*m*×*n*^, each column *x*_*i*_ represents a sample, and generally the LRR uses the data itself as a dictionary. Then the model can be as shown in

(1) $$\begin{array}{c} O_{1}=\left\{\begin{array}{cc} min & {\left\|Z\right\|}_{*}+\lambda {\left\|E\right\|}_{2,1}\\ s.t. & X=XZ+E. \end{array}\right. \end{array}$$

The LRR matrix **Z** = [*z*_1_, *z*_2_, *z*_3_,…, *z*_*n*_] ∈ *ℝ*^*n*×*n*^, and *z*_*i*_ is the linear representation coefficient of the sample *x*_*i*_ under the data dictionary **X**. The original data usually contains a lot of noise, while the sparse constraint can maintain the robustness of the algorithm effectively. Ref. [24] shows the specific solution process of LRR.

Let **Z** = **J**; we construct the following Augmented Lagrangian function:

(2) L=J∗+λE2,1+Λ,Z−J+Π,X−XZ−E+μ2Z−JF2+X−XZ−EF2.

The specific update algorithm is as follows.

Keep **Z** = **Z**^*k*^, Λ = Λ^*k*^; update **J**:

(3) $$\begin{array}{c} J^{k+1}=\underset{J}{arg\;min\;}{\left\|\;J^{k}\;\right\|}_{*}+\frac{\mu^{k}}{2}{\left\|\;\frac{\Lambda^{k}}{\mu^{k}}+Z^{k}-J^{k}\;\right\|}_{F}^{2}. \end{array}$$

Keep **J** = **J**^*k*^,  Λ = Λ^*k*^, and  Π = Π^*k*^; update **Z**:

(4) Zk+1=arg min ZΛkμk+Zk−JkF2+Πkμk+X−XZk−EkF2.

Keep **Z** = **Z**^*k*^, Π = Π^*k*^; update **E**:

(5) Ek+1=arg min EλEk2,1+μk2Πkμk+X−XZk−EkF2.

### 2.2. Lat-LRR

LRR has two conditions; one is that the original data **X** contains enough samples, and the other is that **X** contains enough nonpolluting data. However, these two conditions are almost impossible to achieve for gene data. On the one hand, the available number of gene samples for research is small because of the high prices of gene sequencing. On the other hand, due to process, instrument electromagnetic interference, and other factors, noise pollution will be produced inevitably in the process of genetic sequencing. To overcome the limitation of LRR, [25] proposed a method of Lat-LRR which expressed the original observation data **X** as a linear combination of principal feature **XZ** and latent feature **LX** for feature extraction. Considering the characteristics of heavy noise in gene expression profile, we added sparsity constraints to the model to construct the following Lat-LRR function:

(6) $$\begin{array}{c} O_{2}=\left\{\begin{array}{cc} min & {\left\|Z\right\|}_{*}+{\left\|L\right\|}_{*}+\lambda {\left\|E\right\|}_{2,1}\\ s.t. & X=XZ+LX+E. \end{array}\right. \end{array}$$

The solution of Lat-LRR is given in [26]. Alternating direction method (ADM) is adopted to solve the model (6). Let **Z** = **J**_1_,  **L** = **J**_2_; we constructed the following Augmented Lagrangian function:

(7) L=J1∗+J2∗+λE2,1+Λ,Z−J1+Π,L−J2+Δ,X−XZ−LX−E+μ2Z−J1F2+L−J2F2+X−XZ−LX−EF2.

Keep **Z** = **Z**^*k*^ and  Λ = Λ^*k*^; update **J**_1_:

(8) $$\begin{array}{c} J_{1_{}}^{k+1}=\underset{J_{1}}{arg\;min\;}{\left\|\;J_{1_{}}^{k}\;\right\|}_{*}+\frac{\mu^{k}}{2}{\left\|\;\frac{\Lambda^{k}}{\mu^{k}}+Z^{k}-J_{1_{}}^{k}\;\right\|}_{F}^{2}. \end{array}$$

Keep **L** = **L**^*k*^,  Π = Π^*k*^; update **J**_2_:

(9) $$\begin{array}{c} J_{2_{}}^{k+1}=\underset{J}{arg\;min\;\;}{\left\|\;J_{2}^{k}\;\right\|}_{*}+\frac{\mu^{k}}{2}{\left\|\;\frac{\Pi^{k}}{\mu^{k}}+L^{k}-J_{2}^{k}\;\right\|}_{F}^{2}. \end{array}$$

Keep **J**_1_ = **J**_1_^*k*^,  **L** = **L**^*k*^,  **E** = **E**^*k*^,  Λ = Λ^*k*^, and  Δ = Δ^*k*^; update **Z**:

(10) Zk+1=I+XTX−1·XTX−LkX−Ek+J1k+XTΔk−Λkμk.

Keep **Z** = **Z**^*k*^,  **E** = **E**^*k*^,  Π = Π^*k*^, and  Δ = Δ^*k*^; update **L**:

(11) Lk+1=X−XZk−EkXT+J2k+ΔkXT−Πkμk·I+XXT−1.

Keep **Z** = **Z**^*k*^,  **L** = **L**^*k*^; update **E**:

(12) Ek+1=arg min  EλEk2,1+μk2X−XZk−LkXk−EkF2.

## 3. Method

### 3.1. NNDGLLRR

Lat-LRR overcomes the problem of too many constraints of LRR dictionary; however, Lat-LRR has limited ability to recover the subspace, and too many auxiliary variables are involved in the process of algorithm solving that involves a lot of matrix singularity value decomposition (SVD) and matrix inversion, which will affect the performance of the algorithm. Ref. [27] proposed a feature extraction method combining manifold constraint and nonnegative matrix factorization (NMF). In the case of NMF reducing dimensionality, the internal spatial structure of the data is maintained by manifold regularized constraint, and good experimental results are obtained. Ref. [28, 29] proposed an image clustering method combining manifold regularized constraint with Lat-LRR. Similar to the image data, the gene expression profile is also constituted by numerical matrix with high redundancy and heavy noise. Considering this characteristic, we constructed a new NNDGLLRR model on the basis of the original model.

(13) $$\begin{array}{c} O_{3}=\left\{\begin{array}{cc} \underset{Z}{\min}\; & {\left\|Z\right\|}_{*}+{\left\|L\right\|}_{*}+\frac{\alpha }{2}Tr\left(ZS_{1}Z^{T}\right)+\frac{\beta }{2}Tr\left(LS_{2}L^{T}\right)+\lambda {\left\|E\right\|}_{2,1}\\ s.t. & X=LXZ+E,\;Z\geq 0,\;\;L\geq 0, \end{array}\right. \end{array}$$

where *α*, *β*, and *λ* are nonnegative constants; the model is a nonnegative latent low-rank representation (NNLLRR) when *α* and *β* are equal to zero. Model (13) takes a more general form. The dual regularized constraint is used to preserve the internal spatial structure of the original data, and sparse constraints and nonnegative constraints are used to maintain and enhance the robustness of the algorithm. **S**_1_ and **S**_2_ are Laplacian matrices, **S**_1_ = **D**_1_ − **W**_1_,  **S**_2_ = **D**_2_ − **W**_2_. **W**_1_, and **W**_2_ are weight matrix, and there are many ways to solve **W**, and here we use Gaussian thermal weight. The specific solution is as follows:

(14) W1ij=e−xi−xjF2/σ;i,j=1,2,3,…,nW2ij=e−xiT−xjTF2/σ;i,j=1,2,3,…,m,

where *σ* is a constant; *x*_*i*_ and *x*_*j*_ represent the *i*th column and *j*th column of  **X** (*i*th and *j*th sample); *x*^*i*^ and *x*^*j*^ represent the *i*th row and the *j*th row of  **X**,  **D**_*ij*_ = ∑_*j*_**W**_*ij*_.

ADM is used to solve model (12), and the following augmented Lagrange function is constructed:

(15) L=Z∗+L∗+α2TrZS1ZT+β2TrLS2LT+λE2,1+μ2Λμ+X−LXZ−EF2,

where Λ is a Lagrangian multiplier; *μ* is a constant and *μ* > 0.

Data in real life is generally nonnegative, and nonnegative constraints will make the calculation with a certain degree of sparseness and enhance the robustness of the algorithm. To maintain the nonnegative of feature extraction, we define the following operators:

(16) $$\begin{array}{c} P\left(\;a_{ij}\;\right)=\left\{\begin{array}{cc} a_{ij}; & if\;\;a_{ij}>0\\ 0; & otherwise. \end{array}\right. \end{array}$$

The solution of model (15) is divided into three subproblems: first, the solution of variable **Z**, second, the solution of variables **L**, and, third, the solution variable of  **E**.

*(1) Solving the First Subproblem.* Update **Z**:

(17) Zk+1=arg min  ZZ∗+α2TrZS1ZT+μk2Λkμk+X−LkXZ−EkF2.

Regarding Taylor second-order expansion to (17), the approximate solution of **Z** is as follows:

(18) Zk+1=arg min  ZZ∗+α2TrZS1ZT+ηZμk2Z−Zk−1ηZμkXTLkTΠkF2=arg min  ZZ∗+ηZμk+αS12Z−Zk−1ηZμk+αS1XTLkTΠk−αZkS1F2=D1/ωZZk+1ωZXTLkTΠk−αZkS1.

Nonnegative constraints to **Z** are as follows:

(19) $$\begin{array}{c} {\left(\;Z^{k+1}\;\right)}_{ij}=P{\left(\;Z^{k+1}\;\right)}_{ij}. \end{array}$$

Define *η*_**Z**_ = ∂^2^*h*/∂**Z**^2^;  *h* = (*μ*^*k*^/2)‖Λ^*k*^/*μ*^*k*^ + **X** − **L**^*k*^**XZ**^*k*^ − **E**^*k*^‖_*F*_^2^;  *ω*_**Z**_ = *η*_**Z**_*μ*^*k*^ + *α*‖**S**_1_‖;  Π^*k*^ = Λ^*k*^ + *μ*^*k*^(**X** − **L**^*k*^**XZ**^*k*^ − **E**^*k*^). Ref. [30] gives the solution of *D*_*ε*_(·); the solution process is as follows:

(20) DεφUSεΩVT=arg min  TεΤ∗+12Τ−φF2.

In (20), **UΩV**^*T*^ is the singular value decomposition (SVD) of **φ**, *S*_*ε*_(·) is the vector form of the singular value contraction operator (SVT), and *S*_*ε*_(**Ω**) is defined as follows:

(21) $$\begin{array}{c} S_{\varepsilon }\left(\;\Omega \;\right)=diag\left(\;sgn\left(\;\Omega_{ii}\;\right)\;\right)\left(\;\left|\;\Omega_{ii}\;\right|-\varepsilon \;\right). \end{array}$$

*(2) Solving the Second Subproblem.* Similarly, update  **L**:

(22) Lk+1=arg min  LL∗+β2TrLS2LT+ηLμk2L−Lk−ΠkZkTXTF2=arg min  LL∗+ηLμk+βS22L−Lk−1ηLμk+βS2ΠkZkTXT−βLkS2F2=D1/ωLLk+1ωLΠkZkTXT−βLkS2.

Nonnegative constraints to  **L** are as follows:

(23) $$\begin{array}{c} {\left(\;L^{k+1}\;\right)}_{ij}=P{\left(\;L^{k+1}\;\right)}_{ij}. \end{array}$$

Define *η*_**L**_ = ∂^2^*h*/∂**L**^2^;  *ω*_**L**_ = *η*_**L**_*μ*^*k*^ + *β*‖**S**_2_‖.

*(3) Solving the Third Subproblem.* Update **E**:

(24) Ek+1arg min  EλE2,1+Λk,X−LkXZk−E+μk2X−LkXZk−EF2=λE2,1+μk2Λkμk+X−LkXZk−EF2=Θλ/μkΛkμk+X−LkXZk,

where Θ_*τ*_(·) is a soft threshold operator (ST); Θ_*τ*_(·) is defined as follows:

(25) $$\begin{array}{c} \Theta_{\tau }\left(\;\psi \;\right)=sgn\left(\;\psi \;\right)\max\left(\;\left|\;\psi \;\right|-\tau ,0\;\right). \end{array}$$

The iterative process of each variable of NNDGLLRR is given above. The concrete updating process is shown in Algorithm 1.

### 3.2. Sparse Representation Classifier (SRC)

Sparse representation is a hotspot in the field of pattern recognition in recent years. SRC has been successfully applied in the field of image classification and has achieved relatively ideal experimental results [31]. Similar to the image data, the gene expression profile is also composed by a series of high redundancy and heavy noise of gene samples. In this paper, the latent features extracted by NNDGLLRR are regarded as data dictionary to construct the following SRC model:

(26) $$\begin{array}{c} O_{4}=\underset{\zeta }{arg\;min\;\;}{\left\|\;D\zeta -L^{*}y\;\right\|}_{2}+\gamma {\left\|\;\zeta \;\right\|}_{1}. \end{array}$$

According to the result of SRC, we can get the classification result of unknown gene sample **y**:

(27) $$\begin{array}{c} i^{*}=\underset{i}{arg\;min\;\;}{\left\|\;D\delta_{i}\left(\;\zeta \;\right)-L^{*}y\;\right\|}_{2}. \end{array}$$

The detailed flow of the SRC is shown in Algorithm 2.

### 3.3. Algorithm Flow

To sum up, the algorithm can be divided into two parts; one is to use NNDGLLRR to extract latent features of the original gene expression profile, and the other is to use SRC to classify the latent features. The overall flow is as shown in Algorithm 3.

## 4. Results and Discussion

### 4.1. Selecting the Test Data

To test the feature extraction performance of the algorithm, we used diffuse large B-cell lymphoma [32] (DLBCL), mixed lineage leukemia [33] (MLL), lung cancer [34] (LC), acute lymphoblastic leukemia [35] (ALL) gene sequences to make test, and the sample information of each group of genes as is shown in Table 1.

### 4.2. Accuracy Test

*K*-means and sparse representation classifier (SRC) are simple and common classifiers. To compare the clustering results of *K*-means and SRC, the two kinds of classifiers are used to classify the original gene expression profile. Clustering results are shown in Table 2. It is not difficult to find that the classification effect of SRC is significantly higher than that of *K*-means, which is due to the small number of gene expression profiles. To verify the effectiveness of the algorithm for feature extraction, the extracted features from LRR, Lat-LRR, and NNDGLLRR are classified by SRC. Classification results as shown in Table 2.

Table 2 shows that any one of LRR, Lat-LRR, and NNDGLLRR can achieve feature extraction effectively. However, the feature extraction effect of NNDGLLRR is better than that of Lat-LRR. The category and number of samples, as well as dimension of the gene expression profile, will have an impact on the final recognition effect.

### 4.3. The Influence of Graph Regularized Coefficients

**Figure 1:**
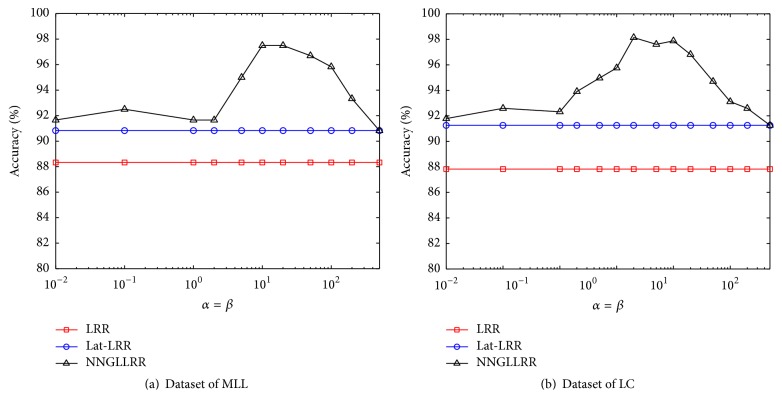
Clustering performance of algorithms with different graph regularized coefficients.

Generally, we set *α* = *β*. To verify the influence of graph regularized coefficients on feature extraction, we have compared the recognition results of LRR, Lat-LRR, and NNDGLLRR under the condition of different *α*  (*β*) values. The results are shown in Figure 1.

Through the test results of MLL and LC, we can find that manifold regularized constraint has obvious optimization effect on the gene expression profile feature extraction when the values of *α* and *β* are appropriate, and it can significantly improve the recognition effect of feature extraction. However, *α* and *β* should not be too large or too small. The optimal graph regularized coefficients may be different for different test data sets.

### 4.4. The Influence of Sparse Representation Coefficients

**Figure 2:**
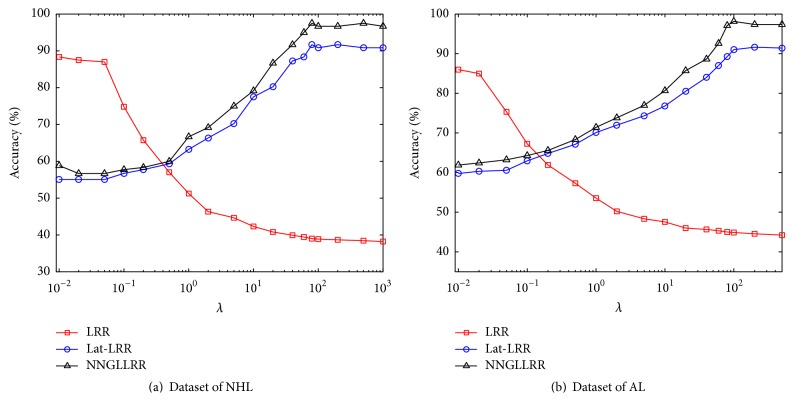
Clustering performance of algorithms with different sparse representation coefficients.

During the process of gene sequencing, the resulting gene expression profile will usually contain heavy noise due to the sequencing process. To verify the effect of the sparse constraint on the feature extraction, we tested the classification accuracy of LRR, Lat-LRR, and NNDGLLRR for feature extraction under different sparse constraint coefficients *λ*. The test results are shown in Figure 2.

Figure 2 shows that different sparse constraint coefficients have a considerable effect on the final feature extraction results. When the value of *λ* is appropriate, the performance of Lat-LRR and NNDGLLRR on feature extraction is better than that of LRR. In general, the performance of NNDGLLRR is better than that of Lat-LRR, which proves the validity of manifold constraint again.

### 4.5. Complexity Analysis

**Z** ∈ *ℝ*^*n*×*n*^,  **L** ∈ *ℝ*^*m*×*n*^, and  **E** ∈ *ℝ*^*m*×*n*^, and we set the lowest ranks of **Z** and **L** obtained by the algorithm as *r*_1_ and *r*_2_. Then the complexity of SVT operation for **Z** and **L** is about **O**(*r*_1_*n*^2^) and **O**(*r*_2_*m*^2^), and the complexity of ST operation for **E** is about **O**(*kmn*). The complexity of construction the Laplacian matrix of **Z** and **L** is about **O**(*mn*^2^) and **O**(*m*^2^*n*); and the complexity of one positive operation for **Z** and **L** is about *n*^2^ and *m*^2^. If the iteration of the algorithm is *k*, then the overall complexity of LRR, Lat-LRR, and NNDGLLRR algorithms is shown in Table 3.

Generally, it is considered that *m* ≫ *n* for gene expression profile. It can be seen from Table 3 that LRR is the simplest in terms of computational complexity, but the performance of LRR on feature extraction is less effective than that of Lat-LRR and NNDGLLRR, and it is difficult to meet the actual demand. The result of Lat-LRR on feature extraction can be not bad, but the partitioning ability of the subspace is limited, and the operation speed is slow because of too many introduced variables. The variable update algorithm of NNDGLLRR not only reduces the calculated amount, but also achieves satisfactory results on feature extraction.

## 5. Conclusion

Aiming at the characteristics of high redundancy and heavy noise of gene expression profile, a feature extraction model of NNDGLLRR is proposed in this paper. In the process of experiment, we extracted the features of different gene expression profile by LRR, Lat-LRR, and NNDGLLRR and classified the extracted features by SRC. The experimental results show that the performance of NNDGLLRR on feature extraction is better than that of LRR and better than Lat-LRR slightly, which verified the comparative advantages of NNDGLRR. At the same time, compared with Lat-LRR, the overall complexity of NNDGLLRR is reduced through the improvement of the variable update algorithm. The experiments using different gene expression data sets for testing have made comparatively ideal experimental results, which proves the validity of the dual graph regularized constraint. In summary, the proposed nonnegative low-rank sparse constraint and dual graph regularized constraint are reasonable, and NNDGLLRR has good adaptability to different gene expression profile with high redundancy and heavy noise.

## Figures and Tables

**Algorithm 1:**
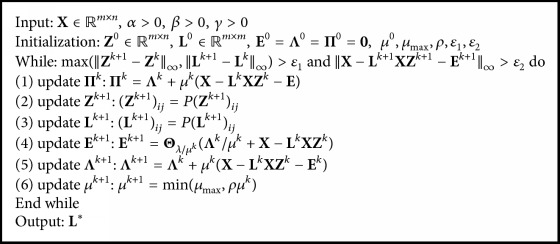
Solving NNDGLLRR model with ALM.

**Algorithm 2:**
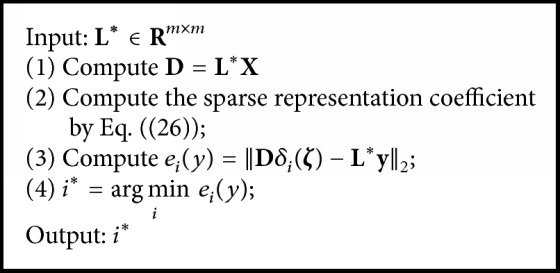
The flow of SRC.

**Algorithm 3:**
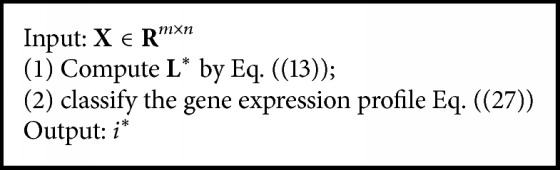
Algorithm flow.

**Table 1:** Test data information.

Data name	Size	Classes	Number
DLBCL	5469 × 77	2	77
MLL	12582 × 72	3	72
LC	12600 × 203	5	203
ALL	12626 × 248	6	248

**Table 2:** Algorithm identification accuracy under different data sets.

Dataset	Accuracy (%)				
	*K*-means	SRC	LRR + SRC	Lat-LRR + SRC	NNDGLLRR + SRC
DLBCL	46.87	69.79	90.62	89.58	94.79
MLL	50.83	71.43	88.33	90.83	97.50
LC	50.79	73.54	87.83	91.26	98.14
AL	43.33	69.44	83.33	87.22	93.32
Average	47.96	71.05	87.53	89.72	95.94

**Table 3:** Algorithm complexity calculation.

	Complexity			
	SVT	ST	Others	Total
LRR	2*kr*_1_*n*^2^	*kmn*	0	**O**(2*kr*_1_*n*^2^ + *kmn*)
Lat-LRR	2*kr*_1_*n*^2^ + 2*kr*_2_*m*^2^	*kmn*	0	**O**(2*kr*_2_*m*^2^)
NNDGLLRR	*kr*_1_ *n* ^2^ + *kr*_2_*m*^2^	*kmn*	*mn* ^2^ + *m*^2^*n* + *kn*^2^ + *km*^2^	**O**(*kr*_2_*m*^2^)
